# The effect of acylation with fatty acids and other modifications on HLA class II:peptide binding and T cell stimulation for three model peptides

**DOI:** 10.1371/journal.pone.0197407

**Published:** 2018-05-14

**Authors:** Heidi S. Schultz, Søren Østergaard, John Sidney, Kasper Lamberth, Alessandro Sette

**Affiliations:** 1 Global research, Novo Nordisk A/S, Måløv, Denmark; 2 La Jolla Institute for Allergy and Immunology, La Jolla, California, United States of America; Hospital Israelita Albert Einstein, BRAZIL

## Abstract

Immunogenicity is a major concern in drug development as anti-drug antibodies in many cases affect both patient safety and drug efficacy. Another concern is often the limited half-life of drugs, which can be altered by different chemical modifications, like acylation with fatty acids. However, acylation with fatty acids has been shown in some cases to modulate T cell activation. Therefore, to understand the role of acylation with fatty acids on immunogenicity we tested three immunogenic non-acylated peptides and 14 of their acylated analogues for binding to 26 common HLA class II alleles, and their ability to activate T cells in an *ex vivo* T cell assay. Changes in binding affinity associated with acylation with fatty acids were typically modest, though a significant decrease was observed for influenza HA acylated with a stearic acid, and affinities for DQ alleles were consistently increased. Importantly, we showed that for all three immunogenic peptides acylation with fatty acids decreased their capacity to activate T cells, a trend particularly evident with longer fatty acids typically positioned within the peptide HLA class II binding core region, or when closer to the C-terminus. With these results we have demonstrated that acylation with fatty acids of immunogenic peptides can lower their stimulatory capacity, which could be important knowledge for drug design and immunogenicity mitigation.

## Introduction

The main function of the immune system is to defend the host from invading pathogens. This defense is mediated by both antibodies and T cells. The T cell response to exogenous proteins relies on a complex series of molecular events. An essential component of this process are major histocompatibility complex class II molecules (MHC II, or HLA Class II in humans), which load short peptides and present them on the surface of APCs. The MHC II:peptide epitope complex can then be recognized by TCR on CD4^+^ T helper cells. If the peptide fragment is of foreign origin, T cells can potentially initiate an immune response. While T cell responses are usually beneficial, particularly in the context of infection, responses to exogenously administered protein drugs may be detrimental and of considerable concern both in terms of safety and efficacy.

Chemical modifications are processes that add to the function and stability of proteins. These protein modifications can be amino acid alterations, such as phosphorylation, methylation or acylation with fatty acid. In drug development, a frequent goal is to prolong the half-life of the compounds. This can be achieved by different methods, such as pegylation, phosphorylation and acylation with fatty acids. However, chemical modifications can also enhance or decrease immunogenicity by altering antigen processing, enhancing or decreasing the capacity of potential epitopes to bind HLA, or modulating TCR recognition [1–4]. Various studies have shown diverse outcomes of conjugating different fatty acids to a diverse range of proteins and peptides. An increase in the immunogenic potential of peptides has been shown for acylation, by e.g. thiopalmitoylation [5, 6]. Moreover, results from vaccine research has shown that acylation with fatty acids can boost the immune response [7, 8]. On the other hand, a decrease in the stimulatory capacity for a peptide linked to palmitic acid has also been observed *in vivo* [9] as well as *in vitro* [10].

The purpose of this study was to systematically assess the effect of acylation with fatty acids on HLA class II:peptide binding and T cell stimulation by examining the role of different fatty acids, their linkers and the position of the acylation. We found that acylation with fatty acids in general had a minor effect on HLA class II:peptide binding, but had a significant effect on T cell stimulation, with stearic acid in particular being associated with reduced peptide immunogenic potential.

## Materials and methods

### Peptide synthesis

The influenza HA and exendin-4 peptides, and unmodified vatreptacog alfa peptide were synthesized and purified at Novo Nordisk A/S (Måløv, Denmark), see S1 Materials and method. The vatreptacog alfa analogues were purchased from Apigenex (Prague, Czech Republic). The exendin-4 and vatreptacog alfa 15-mers were purchased from A and A (San Diego, USA) as purified material on a 1-mg scale. Peptides utilized as radiolabelled ligands were synthesized on larger scale by A and A, and purified (>95%) by reverse phase HPLC.

### Prediction of peptide:HLA binding affinities

Peptide binding affinity for HLA class II alleles was predicted using the IEDB analysis resource tool available at www.iedb.org [11, 12]. The 26 set of HLA reference alleles was analyzed against each peptide and the neural network (NN) algorithm (Consensus or NetMHCIIpan) [13] IC50 (nM) was noted. A low IC50 value indicates a higher affinity.

### Measurement of peptide:HLA binding affinities

Quantitative measurement of peptide binding capacity for the 26 HLA class II molecules to our model peptides was performed in competition assays based on the inhibition of binding of a high affinity radiolabeled peptide to purified MHC molecules as described by Sidney and colleagues [14]. In brief, MHC molecules were obtained from EBV transformed homozygous cell lines or MHC allele-transfected fibroblasts, and purified from cell pellet lysates by repeated passage over protein A Sepharose beads conjugated with specific Abs. Protein purity, concentration and the effectiveness of depletion steps were monitored by SDS-PAGE and BCA assay. The peptide of interest was diluted and co-incubated with a high affinity radiolabeled peptide (0.1–1 nM) at room temperature or 37°C with purified MHC in the presence of a cocktail of protease inhibitors. Following a two-day incubation, MHC bound radioactivity was determined by capturing MHC/peptide complexes on Ab coated (DR: L243, DP: B7/21 and DQ: HB180; all from Sdix, Newark, USA) Lumitrac 600 plates (Greiner Bio-one, Frickenhausen, Germany), and measuring bound cpm using the TopCount (Packard Instrument Co., Meriden, CT) microscintillation counter. The concentration of peptide yielding 50% inhibition of the binding of the radiolabeled peptide was calculated. Under the conditions utilized, where [label]<[MHC] and IC50 ≥ [MHC], the measured IC50 values are reasonable approximations of the true Kd values. Each competitor peptide was tested at six different concentrations covering a 100,000-fold range (30000ug/ml to 0.1ug/ml), and in three or more independent experiments. As a positive control, the unlabeled version of the radiolabeled peptide was tested in each experiment.

### Cells

Peripheral blood samples were obtained from healthy anonymous volunteers of the Region H blood bank (Copenhagen, Denmark) under informed consent, according to the protocol H-D-2008-113 for research use approved by the Danish Scientific Ethical Committee Region Hovedstaden (Legislative Order No. 402 of May 28^th^ 2008). The donors were HLA-typed at HistoGenetics LLC using next generation sequencing (NGS) technology by the means of Illumnia MiSeq (Ossining, NY, USA). The human blood samples (buffy coat from 450ml blood) were processed as followed: PBMCs were purified by Ficoll-Plaque Plus (GE Healthcare, Uppsala, Sweden) density centrifugation. Red blood cells were lysed using RBC lysis buffer (eBioscience, San Diego, USA) and the PBMCs were washed twice in PBS. Average yield was 2-6x10^8^ cells (Nucleocounter NC-200, Sartorius) and viability was 95.4%. Cells were frozen in CLTC-ABC freezing media (C.T.L), according to their protocol, at approximate 30x10^6^cells/ml in CoolCell containers (Biocision).

### Re-stimulation assay

PBMCs were thawed and washed twice in media (RPMI 1640 (Omega) supplemented with 1% pen/strep (Omega), 1% Glutamax (Gemini) and 5% HAS(Life technologies)) containing 2μl/ml benzonase (Millipore, Denmark), then cultured in duplicates at 3×106 cells/well in 24-well plates and stimulated for 14 days with individual peptides or KLH: influenza HA (5μg/ml), exendin-4 (50 μg/ml), vatreptacog alfa peptide (50μg/ml), KLH (30 μg/ml). At days 3, 7 and 10 the media in the wells was replenished with fresh media containing IL-2 (2μg/ml; eBioscience). On day 14 the cells were washed and re-plated on 96-well fluorospot plates in triplicates at 1x10^5^ cells/well. The cells were re-stimulated with corresponding peptides or PHA (10μg/ml) or medium containing 0.125% DMSO (percent DMSO in the pools, as a control). After 20h incubation at 37°C, wells assessed for INF-γ, IL-5 and IL-10 spots.

### Fluorospot

For triple cytokine fluorospot assessment, fluorospot plates were coated with 5μg/ml anti-IFNγ (1-D1K), 5μg/ml anti-IL-5 (TRFK5) and 15μg/ml anti-IL-10 (9D7) for 20 hours at 4°C (all Mabtech). After cell culture the plates were washed with PBS/0.05% Tween 20 and incubated with anti-IFNγ (7-B6-1-FS-FITC), anti-IL-5 (5A10-BAM) and anti-IL-10 (12G8- biotinylated) for 2 hours (All Mabtech). The plates were then washed and incubated with anti-FITC-490, anti-BAM-640 and SA-550 (all 1:200; Mabtech) for 1 hour. Following, the plates were washed and spots developed using fluorescence enhancer for 15 minutes. Spots were counted by computer-assisted image analysis (AID reader). Responses were considered positive if the net spot-forming cells (SFC) per 10^6^ PBMC were >20, the stimulation index>2, and p<0.05 (Student’s t-test, mean of triplicate values of the response against relevant pools or peptides vs. the DMSO control). All samples had a viability >75%, as determined by trypan blue, and reactivity to PHA >400 SFC/10^6^ cells. The total magnitude of response was defined as the mean spot forming cells (SFC) for each individual peptide. The total magnitude of response across participants was defined as the sum of responses per individual.

## Results

### Selection of a panel of peptides and HLA allelic variants for investigation

Three different model polypeptides were chosen to evaluate the effect of acylation with fatty acids on immunogenicity based on their *in vivo* and *in vitro* immunogenic properties (Table 1). These were the Influenza HA (307–319) epitope, the whole exendin-4 synthetic peptide, and a peptide fragment of vatreptacog alfa.

**Table 1:** Model peptides.

	Peptide backbone	Fatty acid	Linker	Conjugation site
Peptide 1	Influenza HA	None	None	
Peptide 2	Influenza HA	C2	Oeg-Oeg-LγGlu	316 (K)
Peptide 3	Influenza HA	C12	Oeg-Oeg-LγGlu	316 (K)
Peptide 4	Influenza HA	C18	LγGlu	316 (K)
Peptide 5	Influenza HA	C18	Oeg-Oeg-LγGlu	316 (K)
Peptide 6	Influenza HA	C18	6x(Oeg)-LγGlu	316 (K)
Peptide 7	Exendin-4	None	None	
Peptide 8	Exendin-4	C18	Oeg-Oeg-LγGlu	N-terminus
Peptide 9	Exendin-4	C18	Oeg-Oeg-LγGlu	12 (K)
Peptide 10	Exendin-4	C18	Oeg-Oeg-LγGlu	20 (WT R to K)
Peptide 11	Exendin-4	C18	Oeg-Oeg-LγGlu	27 (K)
Peptide 12	Exendin-4	C18	Oeg-Oeg-LγGlu	C-terminus
Peptide 13	Exendin-4	2 x C18	Oeg-Oeg-LγGlu	12 (K) + 27 (K)
Peptide 14	Vatreptacog alfa	None	None	
Peptide 15	Vatreptacog alfa	C18	Oeg-Oeg-LγGlu-LγGlu	N-terminus
Peptide 16	Vatreptacog alfa	C18	Oeg-Oeg-LγGlu-LγGlu	300 (WT L to K)
Peptide 17	Vatreptacog alfa	C18	Oeg-Oeg-LγGlu-LγGlu	C-terminus
Truncated 15-mer	Exendin-4 (16–30)	None	None	
Truncated 15-mer	Exendin-4 (16–30)	C18	Oeg-Oeg-LγGlu	27 (K)
Truncated 15-mer	Vatreptacog alfa (295–309)	None	None	
Truncated 15-mer	Vatreptacog alfa (295–309)	C18	Oeg-Oeg-LγGlu	304 (R to K)

Influenza HA (307–319): PKYVKQNTLKLAT

Exendin-4 (1–39): HGEGTFTSDLSKQMEEEAVRLFIEWLKNGGPSSGAPPPS

Vatreptacog alfa (285–309): QLLDRGATALVLQVLNVPRLMTQD

LγGlu: L-gamma-glutamyl, Oeg: 8-amino-3,6-dioxaoctanoic acid, WT: wild-type

The 13 amino acid (aa) influenza HA epitope is a well-recognized immunogenic epitope binding several HLA class II molecules and induces an immunogenic response in approximately 30% of donors [15–17]. Exendin-4 is a 39-aa peptide that shows 53% homology to mammalian glucagon like peptide-1 (GLP-1), and is used for the treatment of diabetes mellitus type 2. It has been shown to elicit an immunogenic response in 40–60% of patients treated with the compound, as well as being a T cell epitope [18–20]. Vatreptacog alfa is a fast-acting recombinant factor VIIa analogue aimed for treating haemophilia patients with inhibitors. The development of vatreptacog alfa was discontinued due to the fact that 11% of treated patients developed anti-drug antibodies (ADAs) against the FVIIa analogue in a phase III study [21]. Here we used a 25-aa peptide that contains two of the three mutations introduced in vatreptacog alfa compared to wild-type FVIIa. It has been suggested that these two mutations are responsible for generation of neo T-cell epitopes contributing to the ADA response towards vatreptacog alfa. Lambert *et al*. characterized this peptide as a HLA class II binder using in silico analysis, measured binding affinities, proteomics analysis and T cell assays [22].

Together, these model peptides were used to explore the effect of acylation with fatty acids on HLA binding capacity and T cell immunogenicity. Different positions of acylation, lengths of fatty acids and lengths of linkers (Oeg’s) were evaluated, altogether generating 17 key peptides with or without acylation with fatty acids.

To assess the HLA class II binding capacity of the model peptides, we screened a panel of 26 different HLA DRB1, DRB3/4/5, DP and DQ allelic variants. This panel of 26 HLA class II molecules is representative of the most common specificities, and combined these molecules provide coverage of approximately 98% of the general worldwide population at the phenotypic level [23, 24].

### Measured and predicted binding affinities of influenza HA, exendin-4 and vatreptacog alfa peptide

In the context of HLA class II binding, it was previously demonstrated that a binding affinity of 1000 nM is a general threshold associated with HLA class II restricted epitopes [25–27]. Here, we used this threshold to define binding of the peptides to HLA-DR alleles.

**Fig 1:**
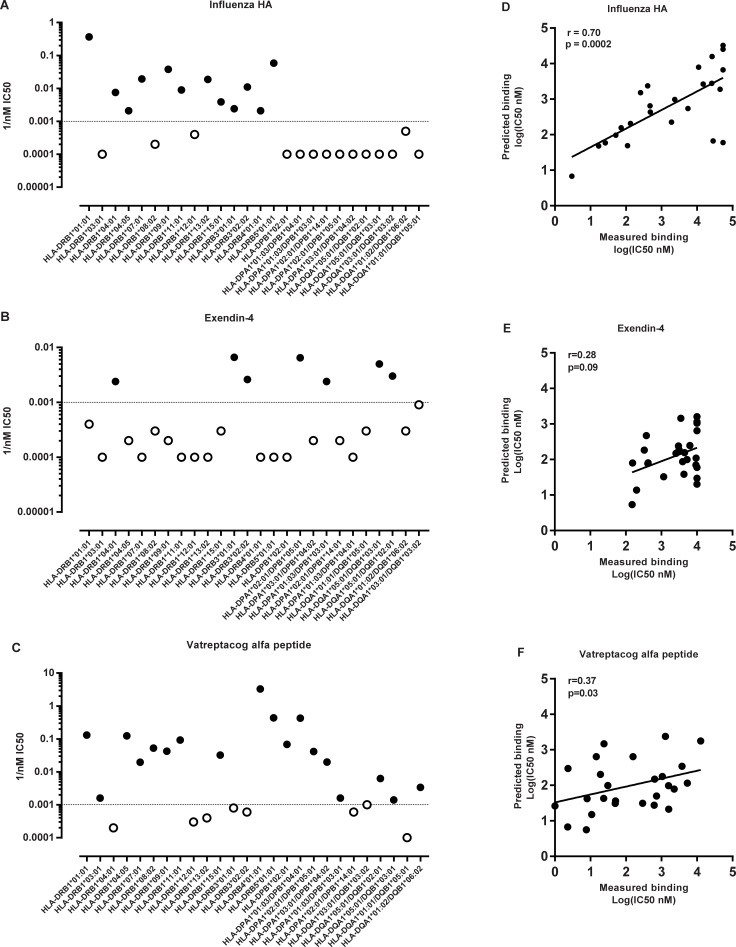
Influenza HA, exendin-4 and vatreptacog alfa peptides binding to HLA class II alleles. Experimental measured binding affinities of (A) Influenza HA, (B) exendin-4 and (C) vatreptacog alfa peptides to 26 alleles. The dotted line indicates IC50 of 1000 nM. The solid black dots indicate alleles that the peptide bind and the open dots indicate non-binding alleles. Comparison of measured and predicted binding data on (D) 13-mer and 15-mer influenza HA epitopes, and (E) exendin-4 and (F) vatreptacog alfa. Affinities are expressed in terms of IC50 nM. The association between the datasets was calculated using a non-parametric two-tailed Spearman rank correlation.

The 13-mer influenza HA peptide is known to bind several different HLA-DR molecules [16, 17, 28–33], and is often utilized as a model HLA DR class II promiscuous binder [32, 34]. We found that 80% (12/15) of the HLA-DR alleles analyzed bound the influenza HA peptide with an affinity of 1000 nM or better (lower IC50 values indicate higher inhibitory capacity and hence better binding affinity). The data is presented in Fig 1A, where 1/nM IC50 is plotted, so that better binding corresponds to a higher value. These data confirm that the 13-mer HA peptide is indeed a promiscuous HLA-DR binder.

Moreover, we compared our measured binding affinities to predicted binding affinities. For this purpose, we applied the suite of HLA class II binding prediction tools available through the immune epitope database (IEDB; www.iedb.org) [11, 12]. Since the IEDB tools require 15-mer sequences as input we performed predictions utilizing a 15-mer version of the HA epitope (CPKYVKQNTLKLATG). Based on previous studies [31] we did not expect significant differences in binding capacity between the 13-mer and 15-mer versions. This assumption was verified by comparing experimentally measured binding affinities of 13-mer and 15-mer influenza HA peptides to the same set of alleles. A good correlation was observed with a r value of 0.91 (data shown in S1 Fig). Hence, the difference in length between the 13-mer and 15-mer does not seem to affect binding to the analyzed alleles. This is probably because the core binding region is the same for the 13- and 15-mer. Predicted IC50 nM for the 15-mer HA peptide was compared to the measured binding data generated for the 13-mer HA peptide and a good correlation was detected with a r value of 0.70 (Fig 1D).

When the binding capacity of intact exendin-4 39-mer was measured experimentally, it was found that it was capable of binding only seven of the 26 HLA molecules tested (Fig 1B), and even in these cases with relatively lower affinity as compared to the predicted affinity of the 15-mers contained in the full-length sequence, which was predicted to be capable of binding 22 out of the 26 alleles (Fig 1E). As the MHC class II peptide-binding groove is open at both ends [35], MHC class II molecules can bind peptides of varying size, usually ranging between 12 and 25 aa. However, while the groove can accommodate long sequences, it is essential that these are available in an extended conformation compatible with binding. It has been demonstrated that Class II molecules can interact with whole denatured proteins, but not native protein antigens [36]. Given that exendin-4 has been shown to fold in a stable conformation, which is associated with its activity as a drug specifically interacting with pancreatic GLP-1 receptors [37–39], this data suggests that the 39-mer’s native configuration limits its capability of binding HLA class II molecules, and that using whole exendin-4 peptide does not seem to be an accurate approach to assess its binding capacity.

In addition, the vatreptacog alfa peptide was evaluated for its capacity to bind the panel of HLA class II molecules. It was found to bind 18 out of the 26 alleles (69%) tested with an affinity of 1000 nM or better, and hence may be considered a promiscuous HLA class II binder (Fig 1C). As also done with the other peptides we compared the measured binding affinities of the vatreptacog alfa peptide to predicted binding affinities. We recorded the best predicted value found for each allele for the entire vatreptacog alfa peptide, which suggest that the vatreptacog alfa peptide has the capacity to bind 23 of the 26 molecules evaluated. However, when comparing the predicted binding data to the measured data we found that the correlation between the two data sets was barely significant (Fig 1F). The relative low correlation was due to the fact that the 25-mer peptide bound fewer alleles than predicted, and several alleles bound less then strongly than predicted. As mentioned above, MHC class II molecules have been shown to preferentially bind peptides in the range of 12–25 aa, and most frequently in the 15–16 residue range. Since the vatreptacog alfa peptide is a 25-mer peptide, it is likely suboptimal in terms of HLA class II:peptide binding capacity, unless further trimming occurs as a result of antigen processing.

### The effect of acylation with fatty acids on HLA binding of the influenza HA epitope

**Fig 2:**
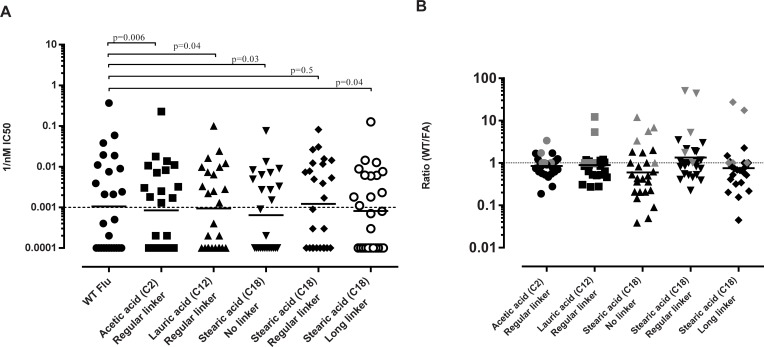
Assessment of binding affinities of acylated and non-acylated influenza HA. (A) Measured binding affinities of influenza HA and acylated analogues of influenza HA. Solid lines show the geometric mean. P-values have been calculated using Wilcoxon t-test. (B) Show changes in binding affinities of non-acylated influenza HA/ acylated influenza HA for each modification. The dotted line indicates 1, which means no change in binding affinities. Numbers above one indicate increased binding as a result of modification, whereas numbers below one indicate decreased binding of the acylated peptides. DQ alleles have been marked with grey. The mean values shown are the geometric mean.

We next examined the effect of acylation with fatty acids on HLA class II binding capacity using five different acylated versions of influenza HA. The different versions included three different fatty acids, namely acetic acid (C2), lauric acid (C12) and stearic acid (C18). All of the fatty acids were attached to a lysine residue at position K316 via a linker that also varied in length. The rationale behind introducing a very short fatty acid (C2) was to assess whether the linker itself had any effect on immunogenicity. The results (Fig 2) show that both the acylated and non-acylated influenza HA could bind HLA class II molecules. However, the average binding affinities for the acylated peptides were in general slightly, but significantly, lower than for non-acylated influenza HA peptide (Fig 2A).

When examining the ratio of binding affinities of the non-acylated peptide compared to the different acylated peptides, we observed that acetic acid and lauric acid had little effect on binding affinities (Fig 2B), where 22 of the 26 (84.6%) and 20 of the 26 (76.9%) affinity measurements were within 2-fold respectively. The binding affinities for stearic acid, irrespective of the linker used, were more variable. Here, most (42–62%) alleles had decreased binding, although several (19–46%) alleles bound the acylated HA peptides better than the non-acylated ones (Fig 2B). Indeed, several alleles showed a consistent pronounced decrease in binding affinities for the acylated peptides (i.e., HLA-DRB1*01:01, HLA-DRB1*09:01 and HLA-DRB1*11:01, HLA-DRB1*13:02 and HLA-DRB5*01:01). Furthermore, we observed that three alleles consistently showed an increased binding of the acylated peptides, namely HLA-DQA1*01:02/B1*06:02, HLA-DQA1*05:01/B1*02:01, and HLA-DQA1 *01:01/B1*05:01 (HLA-DQ alleles marked in grey in Fig 2B). Of particular interest, with respect to unwanted immunogenicity, is that acylation with a fatty acid of the influenza HA peptide changes its HLA-DQA1*01:02/B1*06:02 binding capacity from a non-binder to a binder.

### Assessment of binding affinities of acylated and non-acylated 15-mer exendin-4 and vatreptacog alfa peptide

As described above, the exendin-4 and partially the vatreptacog alfa peptide were not predicted to be optimal, in terms of size, for HLA:peptide binding analysis. For that reason, we decided to synthesize new 15-mer peptides to assess exendin-4 and vatreptacog alfa peptide for binding to the 26 chosen alleles. These 15-mer peptides were chosen based on predictions evaluating the potential binding capacity of 15-mer peptides overlapping by five aa, spanning the entire sequence. For exendin-4, the best and most promiscuous binding was associated with a peptide that covers residues 16–30, which was predicted to bind 16 alleles. For the vatreptacog alfa peptide this was associated with a 15-mer peptide covering residue 295–309, which predict to bind 16 out of 26 alleles (data not shown). Stearic acid acylated analogues were generated.

**Fig 3:**
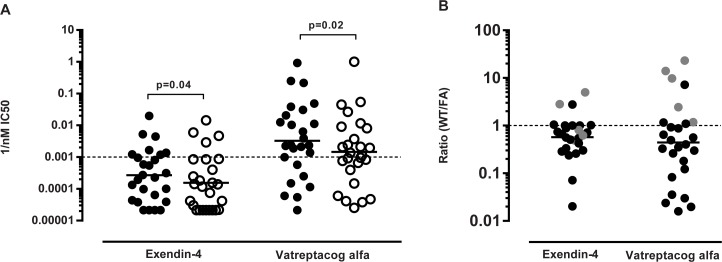
Assessment of binding affinities of acylated and non-acylated exendin-4 and vatreptacog alfa. (A) Measured binding affinities of non-acylated and acylated exendin-4 and vatreptacog alfa 15-mer peptides. Solid dots indicate nonacylated binding affinities and empty dots indicate acylated analogues. (B) Changes in binding affinities plotted as a ratio for 26 alleles. Grey dots indicate DQ alleles. The mean values shown are the geometrical mean.

We next experimentally measured the capacity of these 15-mer peptides, and their respective acylated analogues, to bind the panel of 26 class II alleles. We found that the vatreptacog alfa 15-mer peptide (295–309) was a promiscuous binder, as predicted, binding 18 out of 26 specificities tested. As noted previously, the 25-mer vatreptacog alfa peptide was also measured to bind 18 alleles, and we see an overlap of 15 alleles between the two peptides. The 15-mer exendin-4 (16–30) peptide bound less strongly and less promiscuously (Fig 3), binding 7 of 26, which is less that the 16 alleles it predicts to bind. The predicted and measured binding affinities of vatreptacog alfa and exendin-4 15-mer peptides showed a better correlation (0.51–0.58) than comparing prediction and measured binding affinities for the whole peptides (S2 Fig), which is to be expected since the *in silico* algorithm is primarily based on binding affinities from 15-mer peptides. Nevertheless, in general, we found that the stearic acid-acylated analogues had significantly higher IC50 values (lower binding affinities) than its respective non-acylated counterpart. This suggests that acylation with a stearic acid tends to decrease the binding of a peptide to HLA molecules, in agreement with the data on stearic acid acylated influenza HA. At the same time, also similar to observations with the influenza HA peptide, acylating exendin-4 and vatreptacog alfa peptides seems to increase binding affinity to DQ alleles in general (marked in grey in Fig 3B).

### A study cohort to assess in vitro peptide immunogenicity

**Fig 4:**
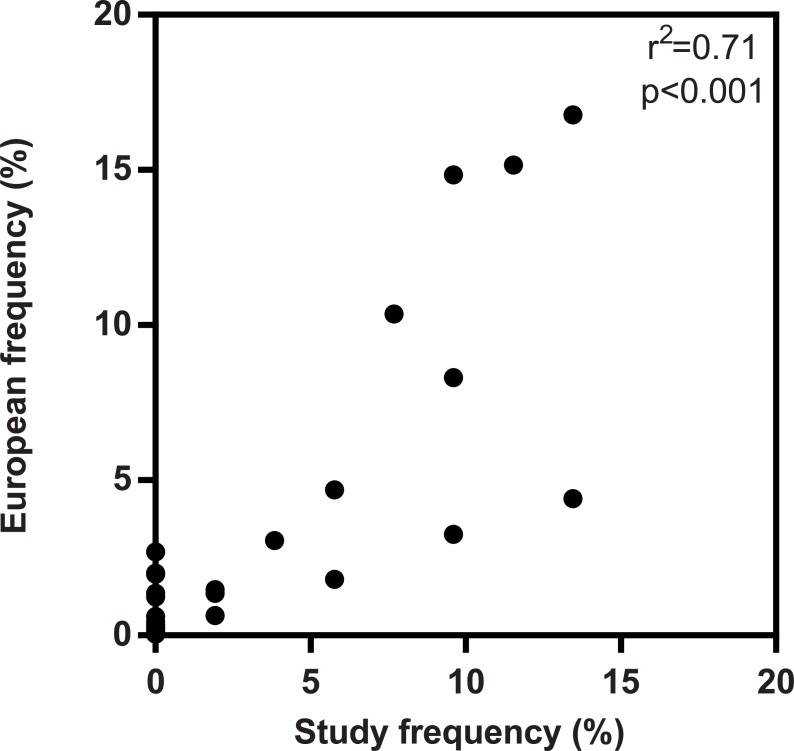
HLA-DRB1 frequency in study population vs. Europeans population. Comparison of the frequency of HLA-DRB1 allotypes expressed in the study cohort versus the European population. The association between the two datasets was calculated using the Pearson rank correlation.

Peptide binding is a condition that is necessary, but not sufficient, for T cell dependent immunogenicity. Accordingly, we wanted to test the effect of acylation with fatty acids on the T cell response. For this purpose, we acquired PBMC donations from 25 healthy volunteers recruited as described in the methods section. All donors included were HLA typed (S1 Table) to verify that the class II alleles expressed in the study cohort provide coverage of the major HLA-DR, DP and DQ allotypes. Further, the allele frequencies of the study population were compared to their distribution in the European population in terms of HLA-DRB1. Average phenotype frequencies for individual DRB1 alleles in the populations are based on data available at allelefrequencies.net [40]. It was noted that HLA frequency distribution in the study population reflects the European population (Fig 4). Since we want to assess how acylation with fatty acids affects responses in a cohort and the alleles are covered by our binding assay panel and should therefore bind a reasonable number of alleles presented by the cohort, our study population appears relevant.

### Evaluation of the effect of linkers and various lengths of fatty acids conjugated to influenza HA on T cell response

T cell responses to the peptides of interest were examined in a two-week re-stimulatory T cell assay using cryopreserved PBMCs from healthy donors. IFN-γ, IL-5 and IL-10 secreting cells were enumerated using a fluorospot apparatus, to determine the frequency of responding donors and the magnitude of the response. These three cytokines were chosen to cover different T helper subsets. IFN-γ is an effector cytokine mainly produced by Th1 cells, whereas IL-5 is predominantly secreted by Th2 cells. IL-10 is considered an anti-inflammatory cytokine primarily secreted by Treg, though it can also be secreted by Th2 cells.

**Fig 5:**
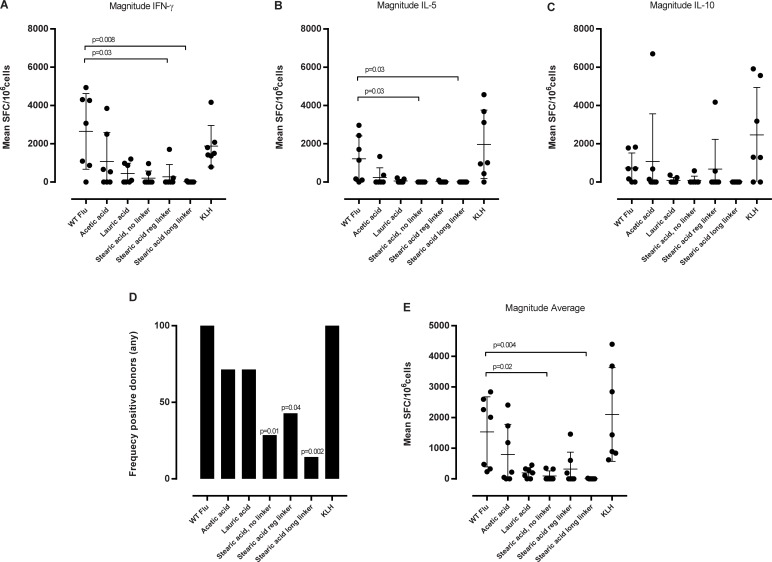
Cytokine secretion upon re-stimulation with non-acylated peptides and their acylated analogues. PBMCs (2x10^6^cells/well) were stimulated with individually peptides (5 μg/ml). At day 3, 7 and 10 the media was replenished and IL-2 added. At day 14 the cells were re-stimulated (5 μg/ml) with their corresponding peptides and respective controls for 20 hours. Cytokine levels for IFN-γ, IL-5 and IL-10 was determined by fluorospot analysis. The figure shows the mean number of spot forming cells (SFC) to (A) IFN-g, (B) IL-5 and (C) IL-10 cytokines for each donor. Significance was calculated in relation to WT influenza HA by one-way ANOVA followed by a Dunn’s multiple comparisons test. (D and E) shows a summary of frequency of responding donors and magnitude of response of all cytokines. (D) Shows the frequency of responding donors to all cytokines (eg one positive hit for one cytokine equals a positive donor) and (E) the corresponding magnitude of the response expressed as an average of spot forming cells (SPC) for all three cytokines. The difference in frequency was calculated using a Fisher’s test and the p-values for the magnitude was calculated in relation to WT influenza HA by one-way ANOVA followed by a Dunn’s multiple comparisons test. Data are shown as mean of seven donors ± SD, each donor are represented by a dot.

Cell cultures stimulated with influenza HA peptide yielded significant numbers of IFN-γ, IL-5 and IL-10 producing cells, and were comparable to the KLH positive control, indicating that the *in vitro* immunization was successful. Influenza HA peptide acylated with a short acetic acid also induced significant numbers of IFN-γ, IL-5 and IL-10 secreting cells with a tendency for lower magnitudes for all three cytokines. In the case of lauric acid- acylation, a weaker response was observed. Acylation with a stearic acid, independent of linker length, led to a drastically reduced cytokine response as seen by the decreased frequency of responding donors, as well as the low magnitude of all three cytokines (Fig 5A–5C) or individual cytokine response (Fig 5D and 5E).

In summary, we observe that conjugation of fatty acids reduces T cell responses towards the influenza HA peptide, and the effects differ as a function of the length of conjugated fatty acid. The longer the fatty acid, the more effective it is in conferring decreased T cell responses, compared to the non-acylated influenza HA peptide.

### Evaluation of the effect on the T cell response of acylation with a C18 stearic acid at different positions throughout the whole exendin-4 peptide

Based on the results shown above for the influenza HA peptide, to address responses to the exendin-4 peptide, we decided to focus only on stearic acid modifications, and by varying the position of steric acid acylation. The 39-aa long polypeptide exendin-4 was modified by conjugating a stearic acid at the N-terminus, position 12 (K), 20 (WT R to K), 27 (K), the C-terminus, and at 12 and 20 simultaneously. At position 20 we introduced a mutation R → K to enable acylation at this position (which has minor effect on predicted binding). All stearic acids were attached with an Oeg-Oeg-LγGlu linker. Preliminary experiments determined that exendin-4 was relatively less immunogenic as compared to the HA peptide, and also determined that 50μg/ml was the optimal concentration for a measurable cytokine response using this assay setup (data not shown).

**Fig 6:**
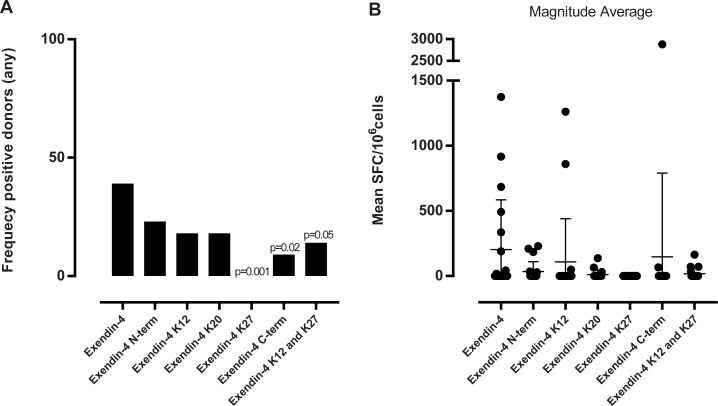
Summary of frequency of responding donors and magnitude of response of all cytokines in all donors. (A) the graph shows the frequency of responding donors to all cytokines (eg one positive hit for one cytokine equals a positive donor) and (B) the graph the corresponding magnitude of the response expressed as an average of spot forming cells (SPC) for all cytokines. n = 22. The difference in frequency was calculated using a one-sided Fisher’s test.

When investigating the cytokine profile of the response of exendin-4 we found that the responding donors mainly secreted all three measured cytokines; IFN-γ, IL-5 and IL-10 (S3 Fig). Non-acylated exendin-4 induced a response in 40% of the donors (Fig 6). Attaching a stearic acid at the N-terminus of exendin-4 mildly decreased the frequency of responding donors, but considerably impacted the magnitude of the response. When the stearic acid was attached at position 12 or 20, further decreases in response frequency, as well as decreased magnitudes were noted. The most pronounced effect of acylation was seen when the stearic acid was conjugated at position 27 or at the C-terminus. None of the donors analyzed responded to exendin-4 acylated with a stearic acid at position 27, and only 10% responded to exendin-4 acylated at the C-terminus. Finally, acylating the peptide at both position 12 and 20 also decreased response frequency and magnitude, although double acylation with fatty acids does not seem to be associated with a more drastic reduction than single acylation.

### Evaluation of the effect of acylation with a C18 stearic acid at different positions throughout the vatreptacog alfa peptide

**Fig 7:**
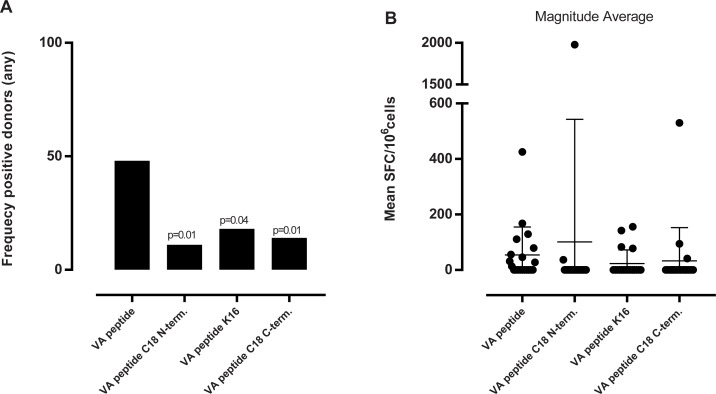
Summary of frequency of responding donors and magnitude of response to the vatreptacog alfa peptide of all cytokines in all donors. (A) the graph shows the frequency of responding donors to all cytokines (eg one positive hit for one cytokine equals a positive donor) and (B) the graph the corresponding magnitude of the response expressed as mean spot forming cells (SFC)/10^6^ cells for all cytokines. n = 22. One-sided Fisher’s test was used to calculate the p value in (A). VA: vatreptacog alfa peptide.

As for exendin-4, titration assays determined that 50μg/ml was also the optimal vatreptacog alfa peptide concentration for stimulating the PBMCs (data not shown). Here, responses, when detected, were generally weak, and mainly associated with IFN-γ and IL-10 secretion (S3 Fig). As shown in Fig 7, non-acylated vatreptacog alfa peptide induced a response in 45% of the donors. Acylated vatreptacog alfa analogues were associated with a lower frequency of responding donors and, with one exception, with corresponding lower response magnitudes.

## Discussion

Acylation with fatty acids of peptides is a known strategy for prolonging peptide half-life, as fatty acids bind albumin, conferring reduce clearance in the kidneys. Examples of acylated peptides with extended half-lives are insulin detemir that has a myristic acid (C14) conjugated to the B29 lysine, and liraglutide, a GLP-1 analogue acylateded with a palmetic acid (C16) [41]. Both compounds were marketed in 2005 and 2009 respectively and have an approved safety profile.

When developing drugs, one major concern is immunogenicity. It has been described that 89% of market compounds report incidences of immunogenicity in the label, where half of these incidences are observed to affect the efficacy of the drug, which is mostly due to the development of ADA’s [42]. Therefore, reducing immunogenicity risk during drug development can be a crucial step in increasing patient safety and drug efficacy. We therefore set out to investigate whether acylation with fatty acids, besides having a positive effect on half-life, also affects HLA binding and T cell responses. Multiple approaches were utilized, including *in silico* peptide:HLA class II binding predicted analysis, *in vitro* peptide:HLA class II binding affinity measurements, and *ex vivo* T-cell activation analyses.

The model peptides chosen for this study was selected due to their specific features as well as being recognized T cell epitopes. Influenza HA is a very well described immunogenic peptide that most likely will generate a secondary response in our donor cohort. Exendin-4 and vatreptacog alfa peptide are more drug-like peptides relevant for drug design as they are low immunogenic, primary response inducing peptides.

We show that HLA class II:peptide binding data correlated well with predicted binding affinities obtained from the iedb.org analysis resource. We also found that large peptides, such as exendin-4, were not suitable for HLA class II:peptide binding analysis, likely due to their inability to be maintained in an extended conformation. Accordingly, for evaluation of the effect of acylation with fatty acids on HLA-class II:peptide binding, we studied the influenza HA peptide and its acylated analogues, as well as selected 15-mer peptides with or without stearic acid acylations derived from the extended full length exendin-4 and vatreptacog alfa sequences.

Using a panel of 26 common HLA class II alleles we found that in the vast majority of cases acylation with fatty acids had a minor effect on MHC binding capacity, yet there was a tendency for acylation with fatty acids to be associated with decreased affinity. Influenza HA peptides conjugated to the shorter fatty acids, acetic acid and lauric acid, were associated with modest decreases in binding affinity, while conjugation with the longer stearic acid was associated with the most prominent decrease in binding affinity. However, certain HLA-DQ alleles were consistently associated with increased binding capacity of the acylated peptides, especially the stearic acid form, and therefore, in some cases, immunogenicity might increase rather than decrease by fatty acid modification. The effect of other chemical modifications, such as citrullination, has previously been shown to a have similarly variable effect on HLA class II binding patterns [43]. That acylation, especially with a stearic acid, shows inconstant effects should be taken into consideration when designing the drug in regards to HLA-DQ alleles. It should therefore be suggested to continue the screening of fatty acids to find one that does not show an increase in binding affinities to any alleles. Nevertheless, taking the T cell response and the HLA-DQ type of the analyzed donors into account, there seems to be no correlation between the specific DQ alleles with increased binding affinities for acylated peptides and some of the few donors there have an enhanced T cell response in respect of those DQ alleles.

To test whether this somewhat variable effect at the level of HLA binding would have a similar counterpart at the level of *in vitro* T cell stimulation, we analyzed the model peptides in a T cell *in vitro* stimulation assay. We found that acylation with fatty acids broadly reduces the T cell response. The acylated analogues of the influenza HA peptide all showed a decrease in frequency of responding donors, particularly when modified with a stearic acid. In respect of magnitude of the response, acylating influenza HA with a short acetic acid (C2) did not induce a significant change in the T cell magnitude, whereas acylation with longer fatty acids like lauric acid (C14) and stearic acid (C18) decreased the magnitude of the response considerably. This indicated that modifications with longer fatty acids are more effective in reducing the T cell response than shorter ones. Related observations on modifications of influenza HA have been made by Hütter et al, where different glycosylations of influenza HA affected T cell activation [44].

When assessing exendin-4 and vatreptacog alfa peptides and their cognate analogues, which carry modifications at different parts of the molecule, we found that the position of the acylation affects the response. Even though all of the acylated versions of exendin-4 had a decreased frequency of responding donors, the most significant effect was observed for exendin-4 acylated at position 27. From our predicted binding analysis we found that the most promiscuous peptide was the 16–30 peptide, where position 27 is part of the core sequence, and therefore it makes good sense that we observe an effect of acylation with a fatty acid at position 27. For the vatreptacog alfa peptide we observed a significant decrease in the stimulatory capacity for all acylated analogues. Since the peptide is only 25 aa long the processing of the peptide is limited and consequently we do not see a more pronounced effect from one of the acylations.

The present study shows that acylation with fatty acids can impact HLA class II binding capacity, although in general the effect is relatively modest, and decreases in binding greater than 10-fold were observed for only a few alleles. Nonetheless, it should be noted that some alleles, especially HLA-DQ alleles, are associated with increased affinity for the acylated analogues. However, *in vitro* T cell experiments showed that acylation with fatty acids of all three model peptides decreased T cell activation, which suggest that acylation with fatty acids is an effective means to decrease immunogenicity and that its mechanism of action is more likely to be mediated by interfering with T cell receptor binding rather than HLA binding. The most noticeable effect of acylation with fatty acids was observed when the stearic acid was positioned within the peptide in the core sequence of a promiscuous epitope.

In conclusion, with this study we have shown that acylation with fatty acids of immunogenic peptides decreases their immunogenic potential, and modulation of immunogenic peptides by acylation with fatty acids could potentially be a favorable approach in drug design as it could decrease undesired immune activation.

For a full understanding of the mechanisms behind the ability of fatty acids to modify the immune response to immunogenic peptides additional experiments could be beneficial. These experiments could include examining earlier events in immune activation, such as antigen uptake and internalization by APCs, as well as HLA:peptide presentation on the cell surface by APCs.

## Supporting information

S1 Fig Comparison of measured and predicted binding data on 13-mer and 15-mer influenza HA epitopes. (PDF)

S2 Fig Comparison of measured and predicted binding affinities for 15-mer exendin-4 and vatreptacog alfa peptides. (PDF)

S3 Fig Cytokine secretion upon re-stimulation with WT exendin-4, vatreptacog alfa peptide and their acylated analogues. (PDF)

S1 Materials and method Synthesis of peptides. (DOCX)

S1 Table High resolution HLA II typing of study cohort. (DOCX)
