# Supplementary material for: The effect of acylation with fatty acids and other modifications on HLA class II:peptide binding and T cell stimulation for three model peptides
Source: PLoS One. 2018 May 14;13(5):e0197407. doi: 10.1371/journal.pone.0197407 (PMC5951580; doi:10.1371/journal.pone.0197407)
Supplement: S1 Fig — (PDF) [file pone.0197407.s001.pdf]

Supplemental data figure 1

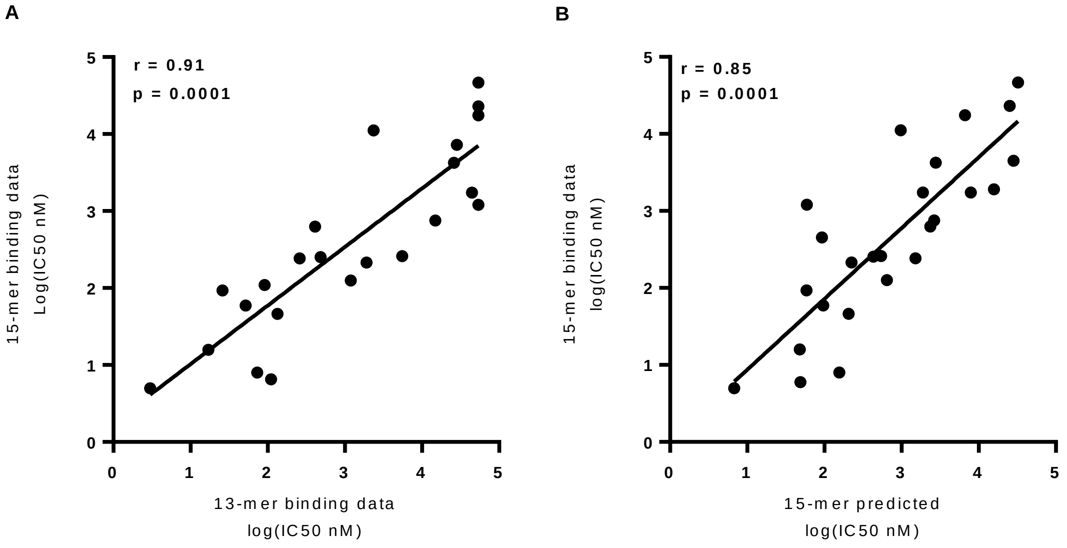

Supplemental figure 1. Comparison of measured and predicted binding data on 13-mer and 15-mer influenza HA epitopes. (**A**) Shown is the correlation between measured binding affinities for the13-mer and 15-mer influenza HA epitopes using 23 class II alleles (0.91). (**B**) The correlation between the measured and predicted affinities using 25 class II alleles for the 15-mer was 0.85. Affinities are expressed in terms of IC50 nM. The association between the datasets was calculated using a non-parametric two-tailed spearman rank correlation.
