# Supplementary material for: The effect of acylation with fatty acids and other modifications on HLA class II:peptide binding and T cell stimulation for three model peptides
Source: PLoS One. 2018 May 14;13(5):e0197407. doi: 10.1371/journal.pone.0197407 (PMC5951580; doi:10.1371/journal.pone.0197407)
Supplement: S2 Fig — (PDF) [file pone.0197407.s002.pdf]

Supplemental data figure 2

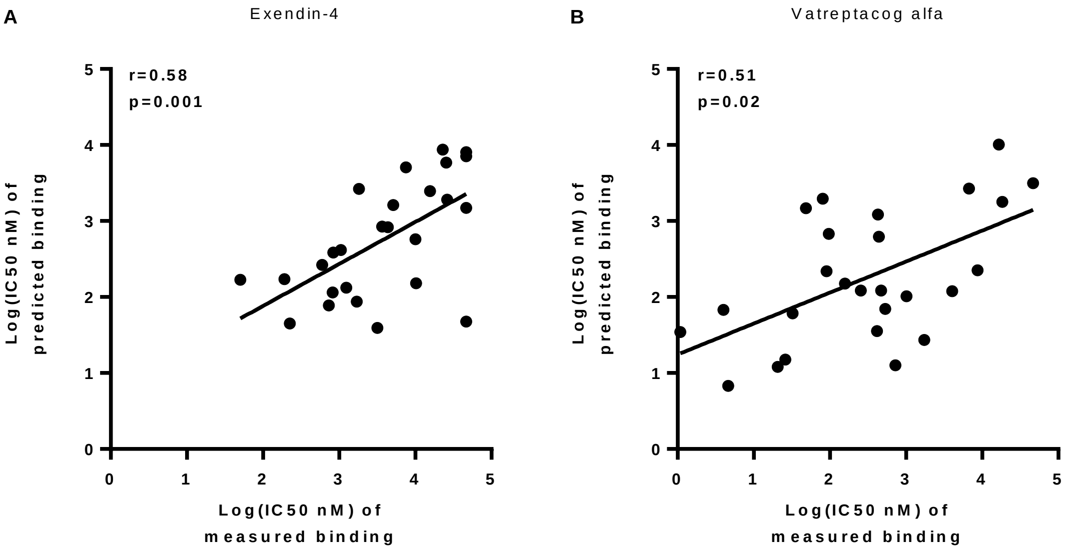

Supplemental figure 2. Comparison of measured and predicted binding affinities for 15-mer exendin-4 and vatreptacog alfa peptides. The correlation between the measured and predicted binding affinities measured using 26 class II alleles was 0.58 for the 15-mer (A) exendin-4 and (B) vatreptacog alfa. The association between the datasets was calculated using a non-parametric one-tailed spearman rank correlation.
