# Supplementary material for: The effect of acylation with fatty acids and other modifications on HLA class II:peptide binding and T cell stimulation for three model peptides
Source: PLoS One. 2018 May 14;13(5):e0197407. doi: 10.1371/journal.pone.0197407 (PMC5951580; doi:10.1371/journal.pone.0197407)
Supplement: S3 Fig — (PDF) [file pone.0197407.s003.pdf]

Supplemental data figure 3

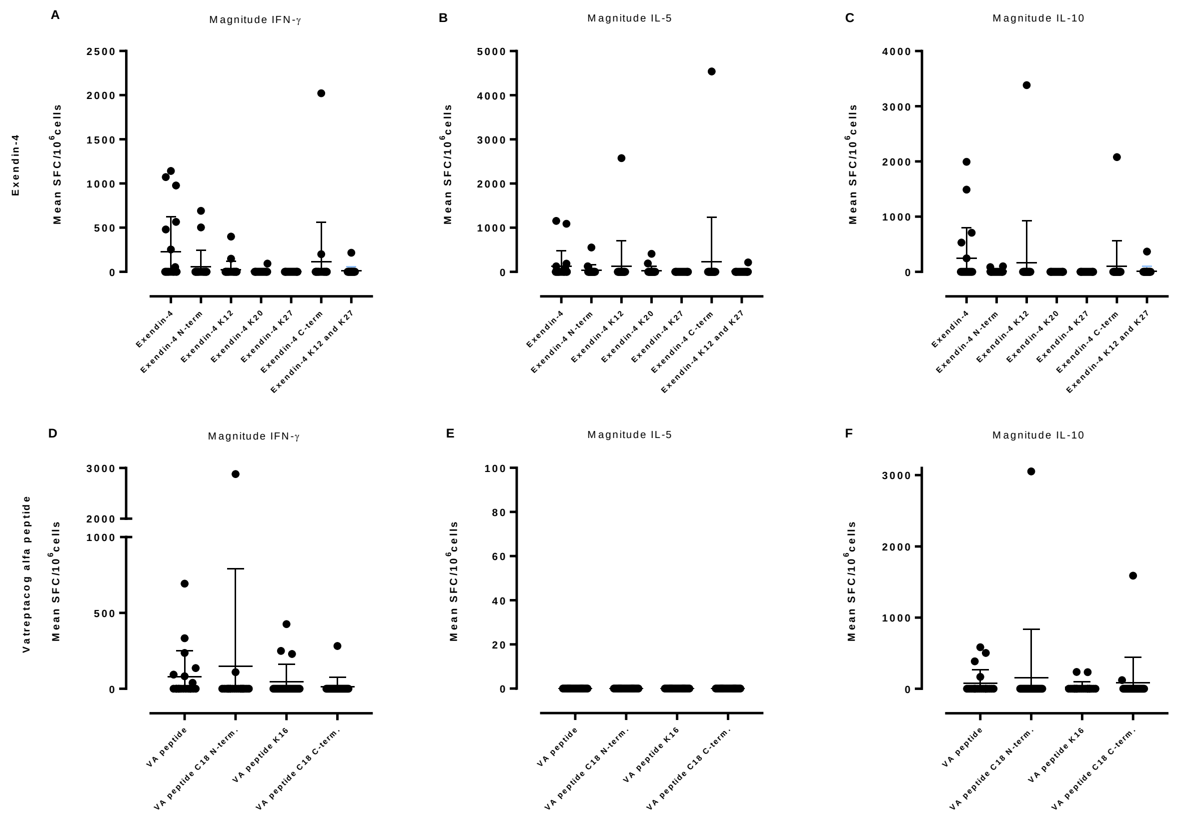

Supplemental figure 3. Cytokine secretion upon re-stimulation with WT exendin-4, vatreptacog alfa peptide and their acylated analogues. PBMCs (2x10^{6}cells/well) were stimulated with individually peptides (50 µg/ml). At day 3, 7 and 10 the media was replenished and IL-2 added. At day 14 the cells were re-stimulated (50 µg/ml) with their corresponding peptides and respective controls for 24 hours. Cytokine levels for IFN-g, IL-5 and IL-10 was determined by fluorospot analysis. The figure shows the mean number of spot forming cells (SFC) to (A, D) IFN-g, (B, E) IL-5 and (C, F) IL-10 cytokines for each donor. Data are shown as mean of 22 donors ± SD, each donor are represented by a dot.
