# Supplementary material for: The effect of acylation with fatty acids and other modifications on HLA class II:peptide binding and T cell stimulation for three model peptides
Source: PLoS One. 2018 May 14;13(5):e0197407. doi: 10.1371/journal.pone.0197407 (PMC5951580; doi:10.1371/journal.pone.0197407)
Supplement: S1 Materials and method — (DOCX) [file pone.0197407.s004.docx]

**Abbreviations:**

ACN: acetonitril

DIC: N,N´-diisopropylcarbodiimide

DCM: dichloromethane

DIPEA: diisopropylethylamine

DMF: N,N-dimethylformamide

FA: formic acid

Fmoc: fluorenylmethyloxycarbonyl

γGlu: L-γ-glutamyl

HFIP: hexafluoroisopropanol

NMP: N-methylpyrrolidone

OEG: 8-amino-3,6-dioxaoctanoic acid

RP-HPLC: reverse phase high-performance liquid chromatography

RP-UPLC: reverse phase ultra-performance liquid chromatography

TFA: trifluoracetic acid

TIPS: triisopropylsilane

*Synthesis of peptides*

All chemicals were of analytical grade or higher. Triisopropylsilane (TIPS), N,N´-diisopropylcarbodiimide (DIC), acetic anhydride, diisopropylethylamine (DIPEA), thioanisole, collidine and formic acid (FA) (≥98%) were from Sigma–Aldrich, Chemie GmBH (Steinheim, Germany). Acetonitrile (ACN), dichloromethane (DCM) (LiChrosolve), trifluoroacetic acid (TFA), and diethyl ether were purchased from Merck KGaA (Darmstadt, Germany). Water came from a MilliQ equipment (Advantage A10) from Millipore (Molsheim, France). 2-Chlorotrityl chloride polystyrene, Rink-amide or PAL polystyrene resins were purchased from Merck KGaA (Darmstadt, Germany). Standard Fmoc amino acids, resins and coupling reagent OxymaPure (Novabiochem®) were from Merck KGaA (Darmstadt, Germany) or Protein Technologies (Tucson, USA). N-Methylpyrrolidone (NMP) dimethylformamide (DMF), hexafluoroisopropanol (HFIP) and piperidine were from Biosolve (Dieuze, France). Fmoc-8-amino-3,6-dioxaoctanoic acid was purchased from Flamma (Italy) and , 18-(tert-butoxy-18-oxooctadecanoic acid, 12-(tert-butoxy-12-oxododecanoic acid were purchase from Syncom (Groningen, The Netherland). Fmoc-L-Glu-Otbu was purchased from Iris Biotech GmbH, (Marktredwitz, Germany)

Peptide analogues were prepared using a Prelude or Prelude X peptide synthesizer (Protein Technologies, Tucson, Arizona US) using a Fmoc-chemistry protocol for solid phase peptide synthesis. Resins used were Fmoc-PAL polystyrene or pre-loaded Wang resin. Each coupling cycle used 5–8 eq. Fmoc-amino acid-OH and OxymaPure in DMF (both 5-8 eq.) and activated by DIC and collidine also both both 5-8 eq. The amino acid solutions were all 0,3M containing also 0,3M OxymaPure. The DIC and collidine solutions were both 3M and added as 1/10 volume compared to the volume of Fmoc-amino acid solutions. For deprotection of Fmoc, 25% piperidine in NMP or DMF was used for 4 + 4 min (or 2+ 2 min using heating 55 ^o^C on Prelude X). Coupling time was set to 60 min (or 30 min using heating 45-55 ^o^C on Prelude X) while double coupling was performed for Fmoc-Arg(Pbf)-OH and a total coupling time of 1 h (or 20 + 40 min using heating 55 ^o^C on Prelude X). For analogues with a side chain Fmoc-Lys(Mtt)-OH was incorporated and the N-terminal of the peptide analogue was Boc-protected. The removal of Mtt was done using HFIP/DCM (3:1) containing 3% TIPS using 3 washes (2 + 2 + 30 min.), and after neutralisation with DIPEA, the side chains was synthesized by standard Fmoc strategy. Peptides were cleaved and deprotected with TFA/TIPS/thioanisole or TFA/TIPS/H_2_O (95:3:2) for 1–3 h and precipitated with diethyl ether. After washing with diethyl ether 4-5 times through a 0.45 μm filter or by centrifugation, the peptides were dried. The peptides were purified by preparative RP-HPLC using a linear gradient: 15-40% ACN with 0.1% TFA over 40 min on a SymmetryPrep C18 19 × 300 mm, 7 μm column (Waters Corporation, Milford, USA) eluting at 20 ml/min. The purity of peptides was determined by analytical RP-UPLC on a Waters Acquity UPLC System with a Waters BEH column C18 using 0.05% TFA in H_2_O (solvent A1) and 0.05% TFA in ACN (solvent B1). Molecular weights were determined using matrix-assisted laser desorption and ionization time-of-flight mass spectroscopy, recorded on a Microflex or Autoflex (Bruker Daltonics, Bremen,Germany). A matrix of α-cyano-4-hydroxy cinnamic acid was used. Alternatively, characterization was performed by UPLC–MS on a setup consisting of a Waters Acquity UPLC system connected to a LCT Premier XE mass spectrometer from Micromass, or by HPLC–MS on an Agilent 1200 series HPLC connected to an Agilent 6230 time-of-flight (TOF) system using solvent A2 and solvent B2 or 0.1% formic acid in H_2_O. The pooled fractions were lyophilized.
