# Supplementary material for: The effect of acylation with fatty acids and other modifications on HLA class II:peptide binding and T cell stimulation for three model peptides
Source: PLoS One. 2018 May 14;13(5):e0197407. doi: 10.1371/journal.pone.0197407 (PMC5951580; doi:10.1371/journal.pone.0197407)
Supplement: S1 Table — (DOCX) [file pone.0197407.s005.docx]

| **Supplemental table 1. High resolution HLA II typing of study cohort** | | | | | | | | |
| --- | --- | --- | --- | --- | --- | --- | --- | --- |
| Donor | **HLA-DRB1** | **HLA-DRB3** | **HLA-DRB4** | **HLA-DRB5** | **HLA-DQB1** | **HLA-DQA1** | **HLA-DPB1** | **HLA-DPA1** |
| 1 | *04:05/*15:01 |  | *01:01 | *01:01 | *03:02/*06:02 | *01:02/*03:01 | *04:01/*04:02 | *01:03/*01:03 |
| 2 | *01:01/*03:01 | *01:01 |  |  | *02:01/*05:01 | *01:01/*05:01 | *02:01/*03:01 | *01:03/*01:03 |
| 3 | *04:01/*13:01 | *01:01 | *01:01 |  | *03:01/*06:03 | *01:03/*03:01 | *02:01/*20:01 | *01:03/*01:03 |
| 4 | *07:01/*15:01 |  | *01:01 | *01:01 | *02:01/*06:02 | *01:02/*02:01 | *04:01/*10:01 | *01:03/*02:01 |
| 5 | *04:01/*10:01 |  | *01:01 |  | *03:02/*05:01 | *01:01/*03:01 | *04:01/*04:01 | *01:03/*01:03 |
| 6 | *12:01/*15:01 | *02:02 |  | *01:01 | *03:01/*06:02 | *01:02/*05:01 | *04:01/*04:02 | *01:03/*01:03 |
| 7 | *08:01/*15:01 |  |  | *01:01 | *04:02/*06:02 | *01:02/*04:01 | *02:01/*04:02 | *01:03/*01:03 |
| 8 | *03:01/*13:01 | *01:01/*02:02 |  |  | *02:01/*06:03 | *01:03/*05:01 | *01:01/*19:01 | *02:01/*02:02 |
| 9 | *04:01/*04:01 |  | *01:02/*01:01 |  | *03:01/*03:01 | *03:01/*03:01 | *11:01/*14:01 | *02:01/*02:01 |
| 10 | *13:01/*15:01 | *01:01 |  | *01:01 | *06:02/*06:03 | *01:03/*01:02 | *03:01/*04:01 | *01:03/*01:03 |
| 11 | *11:01/*13:02 | *02:02/*03:01 |  |  | *03:01/*06:04 | *01:02/*05:01 | *03:01/*04:02 | *01:03/*01:03 |
| 12 | *07:01/*15:01 |  | *01:01 | *01:01 | *02:01/*06:02 | *01:02/*02:01 | *04:01/*04:01 | *01:03/*01:03 |
| 13 | *01:01/*11:04 | *02:02 |  |  | *03:01/*05:01 | *01:01/*05:01 | *04:01/*04:02 | *01:03/*01:03 |
| 14 | *11:03/*15:01 | *02:02 |  | *01:01 | *03:01/*06:02 | *01:02/*05:01 | *01:01/*04:01 | *01:03/*02:02 |
| 15 | *04:01/*12:01 | *02:02 | *01:01 |  | *03:01/*03:02 | *03:01/*05:01 | *04:01/*04:02 | *01:03/*01:03 |
| 16 | *03:01/*15:01 | *01:01 |  | *01:01 | *02:01/*06:02 | *01:02/*05:01 | *03:01/*04:01 | *01:03/*01:03 |
| 17 | *04:01/*08:01 |  | *01:01 |  | *03:01/*04:02 | *03:01/*04:01 | *03:01/*04:01 | *01:03/*01:03 |
| 18 | *01:01/*15:01 |  |  | *01:01 | *05:01/*06:02 | *01:02/*01:01 | *04:01/*04:01 | *01:03/*01:03 |
| 19 | *01:01/*14:01 | *02:01 |  |  | *05:03/*05:01 | *01:01/*01:01 | *04:01/*04:01 | *01:03/*01:03 |
| 20 | *04:01/*15:01 |  | *01:01 | *01:01 | *03:02/*06:02 | *01:02/*03:01 | *02:01/*04:01 | *01:03/*01:03 |
| 21 | *13:01/*15:01 | *01:01 |  | *01:01 | *06:02/*06:03 | *01:03/*01:02 | *04:01/*04:01 | *01:03/*01:03 |
| 22 | *12:01/*13:01 | *02:02/*02:02 |  |  | *03:01/*06:03 | *01:03/*05:01 | *04:01/*04:01 | *01:03/*01:03 |
| 23 | *13:02/*15:01 | *03:01 |  | *01:01 | *06:02/*06:04 | *01:02/*01:02 | *01:01/*03:01 | *01:03/*02:01 |
| 24 | *03:01/*09:01 | *01:01 | *01:01 |  | *02:01/*03:03 | *03:01/*05:01 | *02:01/*04:01 | *01:03/*01:03 |
| 25 | *11:01/*13:01 | *01:01/*02:02 |  |  | *03:01/*06:03 | *01:03/*05:01 | *02:01/*04:01 | *01:03/*01:03 |
